# Alkaline Phosphatase, an Unconventional Immune Protein

**DOI:** 10.3389/fimmu.2017.00897

**Published:** 2017-08-03

**Authors:** Bethany A. Rader

**Affiliations:** ^1^Department of Microbiology, Southern Illinois University, Carbondale, IL, United States

**Keywords:** alkaline phosphatase, hypophosphatasia, tissue non-specific AP, intestinal AP, lipopolysaccharide, microbiome

## Abstract

Recent years have seen an increase in the number of studies focusing on alkaline phosphatases (APs), revealing an expanding complexity of function of these enzymes. Of the four human AP (hAP) proteins, most is known about tissue non-specific AP (TNAP) and intestinal AP (IAP). This review highlights current understanding of TNAP and IAP in relation to human health and disease. TNAP plays a role in multiple processes, including bone mineralization, vitamin B6 metabolism, and neurogenesis, is the genetic cause of hypophosphatasia, influences inflammation through regulation of purinergic signaling, and has been implicated in Alzheimer’s disease. IAP regulates fatty acid absorption and has been implicated in the regulation of diet-induced obesity and metabolic syndrome. IAP and TNAP can dephosphorylate bacterial-derived lipopolysaccharide, and IAP has been identified as a potential regulator of the composition of the intestinal microbiome, an evolutionarily conserved function. Endogenous and recombinant bovine APs and recombinant hAPs are currently being explored for their potential as pharmacological agents to treat AP-associated diseases and mitigate multiple sources of inflammation. Continued research on these versatile proteins will undoubtedly provide insight into human pathophysiology, biochemistry, and the human holobiont.

## Introduction

**Figure 1:**
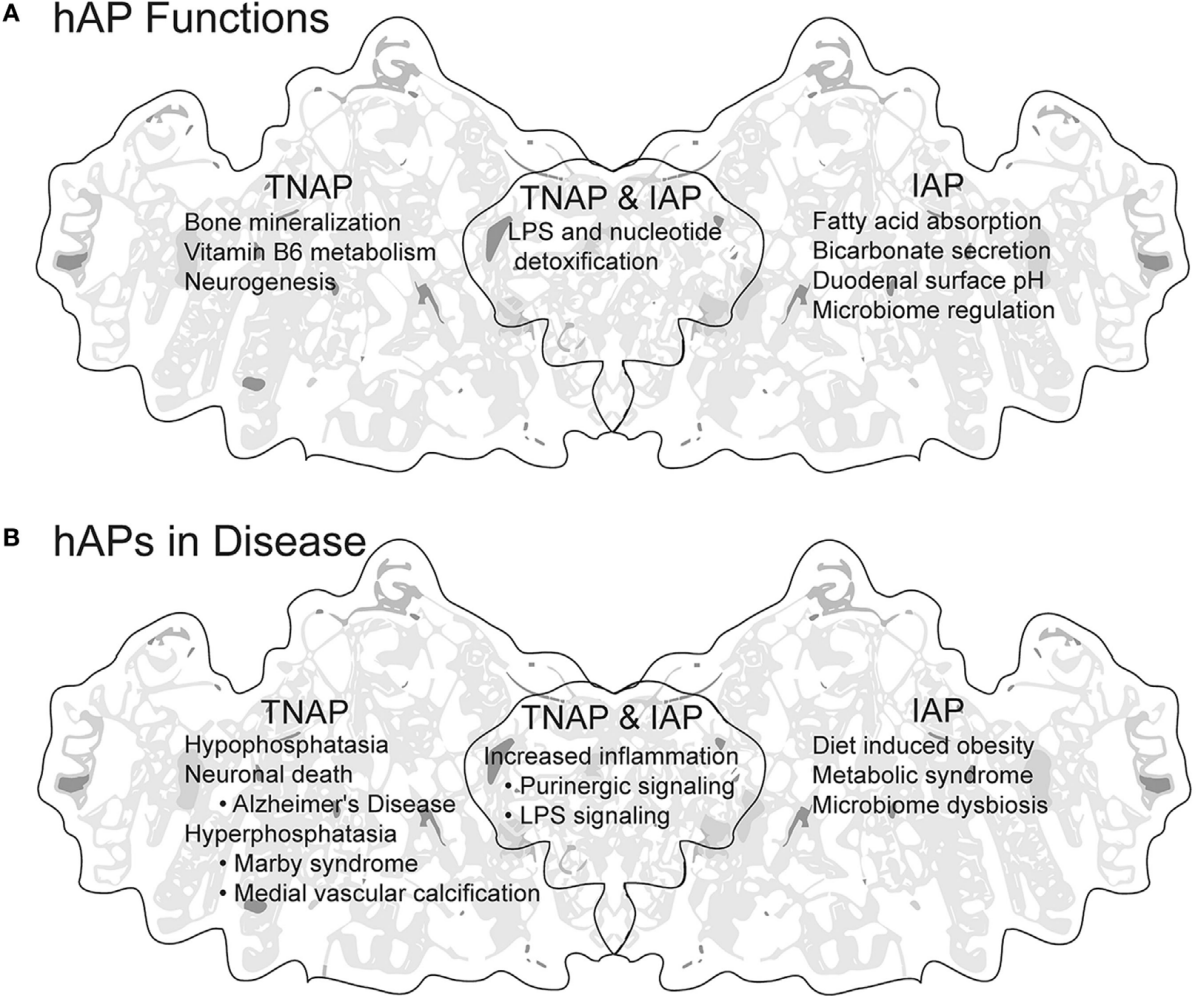
Summary of human APs (hAPs) tissue non-specific AP (TNAP) and intestinal AP (IAP). **(A)** Established and proposed functions of TNAP and IAP. **(B)** Disease states in which increase, decrease, or dysregulation of hAPs is either indicative or causative. Background image modified from the tertiary structure of human PLAP generated by http://www.rcsb.org/pdb (8) PBD ID: 3MK2 (9).

Alkaline phosphatases (APs) belong to a superfamily of proteins (EC 3.1.3.1) sharing conservation of metal binding sites, amino acids required for activity, and predicted fold structure (1). APs are used extensively in life sciences education, as a tool in molecular biology research and as a blood serum marker for liver and bone health, and yet we know surprisingly little about the potential these proteins have to influence our health. In general, APs are anchored to outside surface of the plasma membrane and catalyze the hydrolysis of phosphate groups from a variety of different substrates (dephosphorylation) in an alkaline environment, freeing inorganic phosphate (Pi) (2–4). APs are ubiquitous, with members of the AP super family of proteins extending from the archaea (5) to humans (2). Their ubiquity across life and their expansion and subsequent dynamic evolution in vertebrates implies both variety and conservation of function (6, 7). There are four genes encoding APs in humans. Three genes, *ALPI, ALPP*, and *ALPPL2*, display tissue-specific expression (TSAP proteins), whereas the fourth, *ALPL* is tissue non-specific in expression [tissue non-specific AP (TNAP) proteins] (Table 1). Unlike tissue distribution, surprisingly less is known about the function of these proteins, especially ALPP and ALPPL2 (Table 1). This mini-review will briefly highlight current knowledge of TNAP and intestinal AP (IAP) function in human health and disease (see Figure 1 for summary).

**Table 1:** Description of human alkaline phosphatases (APs).^a^

AP gene	AP protein	Tissue distribution	Known function
*ALPL*	Tissue non-specific AP	Liver, kidney, skeletal tissue, nervous system	Bone and tooth deposition
*ALPP*	PLAP^b^	Syncytiotrophoblasts, reproductive tumors	Unknown
*ALPPL2*	GCAP^b^	Testis, reproductive tumors	Unknown
*ALPI*	IAP^b^	Intestine, enterocyte	Fatty acid absorption, lipopolysaccharide detoxification

*^a^Information from Ref. (2, 7)*.

*^b^TSAPs*.

## Tissue Non-Specific AP

The most direct link between APs and human disease is hypophosphatasia (HPP), a disease characterized by mutations in TNAP associated with decreased enzyme activity in specific organs (10, 11) (Figure 1B). This decrease in AP activity results in variable symptoms that range from perinatal HPP that can result in still birth from profound skeletal hypomineralization (11, 12), potentially lethal seizures in infantile HPP (13–15), to milder phenotypes such as bone fractures and periodontal disease in juvenile HPP and adult HPP (16, 17). A relatively recent mouse model for HPP, in conjunction with medical data and genetic analysis has provided insight into the mechanism of HPP pathophysiology regarding at least two TNAP substrates, extracellular pyrophosphate (PPi), and pyridoxal-5-phosphate (PLP) (7).

## Hypophosphatasia

Tissue non-specific AP is anchored to the cell membranes of osteoblasts and chondrocytes and to matrix vesicles released by those cells, where it degrades PPi to Pi. PPi is an inhibitor of mineralization (18) and regulation by TNAP controls propagation of extracellular mineralization of apatite crystals. TNAP deficiency increases the amount of inhibitory PPi thus decreasing extracellular mineralization, and humans with HPP show a loss of mineralization fronts (19). This has been recapitulated in a TNAP knockout mouse model for infantile HPP (20–22). The loss of mineralization results in various symptoms including softening of bone, bowing and spontaneous breakage of bones, rickets, and tooth (dentin/cementum/enamel) defects (23).

Pyridoxal-5-phosphate, the active form of vitamin B6 (24), is elevated in the serum of HPP patients (25, 26). Hydrolysis of PLP to pyridoxal (PL) by TNAP facilitates diffusion of PL across cell membranes, where it is then re-phosphorylated into PLP. PLP is a versatile cofactor for an estimated 4% of enzymatic reactions and is used by over 110 enzymes to produce or metabolize various molecules (27). PLP-dependent enzymes in the brain are responsible for the production of important neurochemicals including serotonin, dopamine, and gamma-aminobutyric acid (28). The decrease in PLP and resulting decrease in PLP-dependent metabolism in the brain in perinatal HPP patients has been implicated as the cause of neonatal seizures (29, 30).

## Non-HPP TNAP Pathophysiology

Tissue non-specific AP has been implicated in non-HPP related medical conditions (Figure 1B). TNAP is expressed during embryonic neural and spinal chord development, and promotes axonal growth *in vitro* and neurogenesis in adults (31), suggesting an importance in proper neural function. Indeed, increased TNAP activity in the brain has been demonstrated in postmortem hippocampus and serum samples from Alzheimer’s disease patients and has been implicated in neuronal death through increased dephosphorylation of tau (32). Increased serum levels of AP (TNAP and/or TSAPs) due to mutations in GPI anchor synthesis, termed hyperphosphatasia, results most notably in Marby syndrome characterized by seizures, intellectual disability, and facial dysmorphology (33). TNAP upregulation in the vasculature contributes to medial vascular calcification causing vascular stiffening and eventually heart failure (34, 35). An emerging function for TNAP is regulation of purinergic signaling. Extracellular ATP and ADP, through the binding of nucleotide receptors, act as signals inducing inflammation after an acute event such as necrosis induced by damage or infection that releases intracellular nucleotides. In contrast, degradation of extracellular ATP and ADP to AMP and adenine causes cessation of inflammatory signaling, and induction through adenine receptors of an anti-inflammation response (36, 37). TNAP has been implicated in protection against inflammation in multiple diseases and promotion of intestinal microbial populations through hydrolysis of extracellular ATP/ADP to AMP and adenosine (38–40).

## Intestinal AP

Intestinal AP is expressed in villus-associated enterocytes where it regulates fatty acid absorption through secretion of vesicles at both the luminal and basolateral surfaces (41, 42), regulates bicarbonate secretion and duodenal surface pH (43), and has been implicated in the regulation of diet-induced obesity (44, 45) and metabolic syndrome (46, 47) (Figure 1A). But perhaps, the most remarkable function of IAP centers on its protective interactions with the bacterial symbionts that inhabit or invade our enteric system. IAP has been shown to dephosphorylate (detoxify) the lipid A moiety of lipopolysaccharide (LPS), the outer lipid layer of the outer membrane of Gram-negative bacteria (48). In vertebrates, these phosphates are important for binding of LPS to the toll-like receptor 4/MD-2 innate immune receptor complex (49), initiation of NF-kB signaling, and immune response induction (50–52).

Intestinal AP deficiency has been associated with inflammation in the human intestine (53) and in the intestines of vertebrate models in which AP levels are decreased (54). Supplementation of IAP to animals where intestinal inflammation is induced directly or indirectly (with antibiotic use for example) reduces inflammation (53, 55, 56). In addition, a protective role has been ascribed to IAP in mouse models of necrotizing enterocolitis (57–59). This protective role may include IAP-dependent shaping (60) and homeostasis (61) of the microbiome. Along with direct regulation of intestinal homeostasis, IAPs and LPS detoxification have been implicated in other immune-related processes including prevention of bacterial translocation by endogenous or pharmacologically administered IAPs (62–64), and resolution of intestinal inflammation and tissue regeneration (65–67). It should also be noted that in addition to vertebrate IAP, TNAP has been shown to dephosphorylate LPS when it is applied to tissue sections from rat livers (68) and in the mouse uterus (69). With the current and increasing interest in the microbiome, IAP function as it relates to interaction with the endogenous microbes and its influence on human health will undoubtedly be clarified in the coming years.

## Clinical Use of APs

Although there are a multitude of AP studies focusing on vertebrate models of disease, there are relatively few publications to date reporting pharmacological use of APs as a treatment in humans. At the time this article was written, a search of http://clinicaltrials.gov using AP as a search term produced over several hundred responses, however, the vast majority assay for AP levels in serum (a constant hazard when searching any science or medical database using “alkaline phosphatase” as a search term). However, there were at least 11 clinical trials concerning AP treatment of HPP, 3 concerning AP treatment of sepsis with renal injury or failure, 2 concerning AP treatment during or after cardiac surgery, and at least 1 each concerning AP treatment of rheumatoid arthritis, and ulcerative colitis (UC). Interestingly, these studies use several AP sources such as isolated bovine IAP (bIAP), recombinant bIAP, and recombinant human Aps (hAPs). AP enzyme replacement therapy is also currently available to treat HPP. A recombinant soluble human TNAP has been approved for use in perinatal, infantile, and juvenile-onset HPP (70, 71) and has proven successful in symptom improvement and survival in perinatal and infantile HPP (72, 73). In addition to HPP, use of AP as treatment increased renal function in sepsis-induced acute kidney injury (74, 75) and showed short-term improvement of severity of UC in patients with moderate-to-severe UC (76). These studies are a first glimpse into AP use as a treatment for disease, with very positive results. Given the jack of all trades nature of APs and the potential for APs as pharmacological agents in various diseases, studies like these should increase in the coming years.

## Perspective

The ability of APs to detoxify LPS appears to be an evolutionarily conserved function as it was recently implicated in symbiont recognition and homeostasis in the invertebrate squid-*Vibrio* symbiosis model (77). As it is becoming clear that metazoans developed in a microbial world (78), it seems likely that APs have been and may continue to be an evolutionary force shaping the diversity and function of our endogenous microbial populations. Indeed, alterations in IAP have been shown to influence the composition of the intestinal microbiome (60). We can even expand this thinking—if hAPs evolved from an ancient ancestral bacterial AP, then APs may have had a prominent role in shaping basic human biochemistry in addition to our interactions with microbes, and thus exerted a profound influence on human health.

The reader of this review will notice that many of the articles cited might be considered old, with contributions from the 1960s, 1970s, and 1980s. In fact, the study of APs goes back close to 100 years when a bone enzyme freeing phosphate was first mentioned by Robison and Soames (79). That begs the question: how is it, after 90+ years, we still know relatively little about the overall functions of APs? The recent resurgence of interest in APs, should it continue, will hopefully provide more insight into all aspects of AP biology, especially as it relates to health. The ubiquity and functions of AP distinguish them as unconventional immune proteins, and to this writer, APs are unendingly fascinating.

## Author Contributions

BR solely contributed to the production of this manuscript.

## Conflict of Interest Statement

The author declares that the research was conducted in the absence of any commercial or financial relationships that could be construed as a potential conflict of interest.

## References

[1] Galperin MY Koonin EV Bairoch A. A superfamily of metalloenzymes unifies phosphopentomutase and cofactor-independent phosphoglycerate mutase with alkaline phosphatases and sulfatases. Protein Sci (1998) 7:1829–35. 10.1002/pro.5560070819 10082381 PMC2144072

[2] Millán JL Mammalian Alkaline Phosphatases: From Biology to Applications in Medicine and Biotechnology. Weinheim: Wiley-VCH (2006).

[3] Lallès JP. Intestinal alkaline phosphatase: multiple biological roles in maintenance of intestinal homeostasis and modulation by diet. Nutr Rev (2010) 68:323–32. 10.1111/j.1753-4887.2010.00292.x 20536777

[4] Lallès J-P Intestinal alkaline phosphatase: novel functions and protective effects. Nutr Rev (2014) 72:82–94. 10.1111/nure.12082 24506153

[5] Zimmerman AE Martiny AC Allison SD. Microdiversity of extracellular enzyme genes among sequenced prokaryotic genomes. ISME J (2013) 7:1187–99. 10.1038/ismej.2012.176 23303371 PMC3660669

[6] Yang Y Wandler AM Postlethwait JH Guillemin K. Dynamic evolution of the LPS-detoxifying enzyme intestinal alkaline phosphatase in zebrafish and other vertebrates. Front Immunol (2012) 3:314. 10.3389/fimmu.2012.00314 23091474 PMC3469785

[7] Buchet R Millán JL Magne D. Multisystemic functions of alkaline phosphatases. Methods Mol Biol (2013) 1053:27–51. 10.1007/978-1-62703-562-0_3 23860646

[8] Berman HM Westbrook J Feng Z Gilliland G Bhat TN Weissig H The protein databank. Nucleic Acids Res (2000) 28:235–42. 10.1002/0470020571.ch10 10592235 PMC102472

[9] Stec B Cheltsov A Millán JL. Refined structures of placental alkaline phosphatase show a consistent pattern of interactions at the peripheral site. Acta Crystallogr Sect F Struct Biol Cryst Commun (2010) 66:866–70. 10.1107/S1744309110019767 20693656 PMC2917279

[10] Linglart A Biosse-Duplan M. Hypophosphatasia. Curr Osteoporos Rep (2016) 14:95–105. 10.1007/s11914-016-0309-0 27084188

[11] Mornet E Hypophosphatasia: the mutations in the tissue-nonspecific alkaline phosphatase gene. Hum Mutat (2000) 15:309–15. 10.1002/(SICI)1098-1004(200004)15:4<309:AID-HUMU2>3.0.CO;2-C 10737975

[12] Olech EM Zemojtel T Sowińska-Seidler A Robinson PN Mundlos S Karczewski M Identification of a molecular defect in a stillborn fetus with perinatal lethal hypophosphatasia using a disease-associated genome sequencing approach. Pol J Pathol (2016) 67:78–83. 10.5114/pjp.2016.59480 27179278

[13] Baumgartner-Sigl S Haberlandt E Mumm S Scholl-Bürgi S Sergi C Ryan L Pyridoxine-responsive seizures as the first symptom of infantile hypophosphatasia caused by two novel missense mutations (c.677T > C, p.M226T; c.1112C > T, p.T371I) of the tissue-nonspecific alkaline phosphatase gene. Bone (2007) 40:1655–61. 10.1016/j.bone.2007.01.020 17395561

[14] Nunes ML Mugnol F Bica I Fiori RM. Pyridoxine-dependent seizures associated with hypophosphatasia in a newborn. J Child Neurol (2002) 17:222–4. 10.1177/088307380201700314 12026240

[15] De Roo MG Abeling NG Majoie CB Bosch AM Koelman JH Cobben JM Infantile hypophosphatasia without bone deformities presenting with severe pyridoxine-resistant seizures. Mol Genet Metab (2014) 111:404–7. 10.1016/j.ymgme.2013.09.014 24100244

[16] Moulin P Vaysse F Bieth E Mornet E Gennero I Dalicieux-Laurencin S Hypophosphatasia may lead to bone fragility: don’t miss it. Eur J Pediatr (2009) 168:783–8. 10.1007/s00431-008-0835-6 18818947

[17] Weber TJ Sawyer EK Moseley S Odrljin T Kishnani PS Burden of disease in adult patients with hypophosphatasia: results from patient-reported outcome surveys. Metabolism (2014) 65:1522–30. 10.1016/j.metabol.2016.07.006 27621187

[18] Fleisch H Bisaz S Mechanism of calcification: inhibitory role of pyrophosphate. Nature (1962) 195:911 10.1038/195911a0 13893487

[19] Anderson HC Hsu HH Morris DC Fedde KN Whyte MP. Matrix vesicles in osteomalacic hypophosphatasia bone contain apatite-like mineral crystals. Am J Pathol (1997) 151:1555–61. 9403706 PMC1858375

[20] Goldberg RF Austen WG Zhang X Munene G Mostafa G Biswas S Intestinal alkaline phosphatase is a gut mucosal defense factor maintained by enteral nutrition. Proc Natl Acad Sci U S A (2008) 105:3551–6. 10.1073/pnas.0712140105 18292227 PMC2265168

[21] Fedde KN Blair L Silverstein J Coburn SP Ryan LM Weinstein RS Alkaline phosphatase knock-out mice recapitulate the metabolic and skeletal defects of infantile hypophosphatasia. J Bone Miner Res (1999) 14:2015–26. 10.1359/jbmr.1999.14.12.2015 10620060 PMC3049802

[22] Millán JL Yadav M Simao A Narisawa S Huesa C McKee MD Loss of bone mineralization by the simultaneous ablation of PHOSPHO1 and alkaline phosphatase function. Bone (2010) 46:S78 10.1016/j.bone.2010.01.191 PMC3179344 20684022

[23] Millán JL. The role of phosphatases in the initiation of skeletal mineralization. Calcif Tissue Int (2013) 93:299–306. 10.1007/s00223-012-9672-8 23183786 PMC3594124

[24] Shideler CE. Vitamin B6: an overview. Am J Med Technol (1983) 49:17–22. 6342384

[25] Whyte MP Mahuren JD Vrabel LA Coburn SP Markedly increased circulating pyridoxal-5’-phosphate levels in hypophosphatasia. Alkaline phosphatase acts in vitamin B6 metabolism. J Clin Invest (1985) 76:752–6. 10.1172/JCI112031 4031070 PMC423894

[26] Whyte MP Mahuren JD Fedde KN Cole FS McCabe ERB Coburn SP Perinatal hypophosphatasia: tissue levels of vitamin B6 are unremarkable despite markedly increased circulating concentrations of pyridoxal-5’-phosphate. Evidence for an ectoenzyme role for tissue-nonspecific alkaline phosphatase. J Clin Invest (1988) 81:1234–9. 10.1172/JCI113440 3350970 PMC329654

[27] Percudani R Peracchi A. The B6 database: a tool for the description and classification of vitamin B6-dependent enzymatic activities and of the corresponding protein families. BMC Bioinformatics (2009) 10:273. 10.1186/1471-2105-10-273 19723314 PMC2748086

[28] Cruz T Gleizes M Balayssac S Mornet E Marsal G Millán JL Identification of altered brain metabolites associated with TNAP activity in a mouse model of hypophosphatasia using untargeted NMR-based metabolomics analysis. J Neurochem (2017) 140:919–40. 10.1111/jnc.13950 28072448 PMC5339068

[29] Sebastian-Serrano A Engel T de Diego-Garcia L Olivos-Ore LA Arribas-Blazquez M Martinez-Frailes C Neurodevelopmental alterations and seizures developed by mouse model of infantile hypophosphatasia are associated with purinergic signalling deregulation. Hum Mol Genet (2016) 1–14. 10.1093/hmg/ddw248 27466191 PMC5291194

[30] Balasubramaniam S Bowling F Carpenter K Earl J Chaitow J Pitt J Perinatal hypophosphatasia presenting as neonatal epileptic encephalopathy with abnormal neurotransmitter metabolism secondary to reduced co-factor pyridoxal-5′-phosphate availability. J Inherit Metab Dis (2010) 33:S25–33. 10.1007/s10545-009-9012-y 20049532

[31] Zimmermann H Langer D. Tissue-nonspecific alkaline phosphatase in the developing brain and in adult neurogenesis. Subcell Biochem (2015) 76:61–84. 10.1007/978-94-017-7197-9_4 26219707

[32] Kellett KAB Hooper NM The role of tissue non-specific alkaline phosphatase (TNAP) in neurodegenerative diseases: Alzheimer’s disease in the focus. Subcell Biochem (2015) 76:363–74. 10.1007/978-94-017-7197-9_17 26219720

[33] Cole DEC Thompson MD. Neurogenetic aspects of hyperphosphatasia in Mabry syndrome. Subcell Biochem (2015) 76:343–61. 10.1007/978-94-017-7197-9_16 26219719

[34] Sheen CR Kuss P Narisawa S Yadav MC Nigro J Wang W Pathophysiological role of vascular smooth muscle alkaline phosphatase in medial artery calcification. J Bone Miner Res (2015) 30:824–36. 10.1002/jbmr.2420 25428889 PMC4406354

[35] Savinov AY Salehi M Yadav MC Radichev I Millán JL Savinova OV. Transgenic overexpression of tissue-nonspecific alkaline phosphatase (TNAP) in vascular endothelium results in generalized arterial calcification. J Am Heart Assoc (2015) 4. 10.1161/JAHA.115.002499 26675253 PMC4845279

[36] Idzko M Ferrari D Eltzschig HK. Nucleotide signalling during inflammation. Nature (2014) 509:310–7. 10.1038/nature13085 24828189 PMC4222675

[37] Cauwels A Rogge E Vandendriessche B Shiva S Brouckaert P. Extracellular ATP drives systemic inflammation, tissue damage and mortality. Cell Death Dis (2014) 5:e1102. 10.1038/cddis.2014.70 24603330 PMC3973196

[38] Malo MS Moaven O Muhammad N Biswas B Alam SN Economopoulos KP Intestinal alkaline phosphatase promotes gut bacterial growth by reducing the concentration of luminal nucleotide triphosphates. Am J Physiol Gastrointest Liver Physiol (2014) 306:G826–38. 10.1152/ajpgi.00357.2013 24722905 PMC4024727

[39] Peters E Geraci S Heemskerk S Wilmer MJ Bilos A Kraenzlin B Alkaline phosphatase protects against renal inflammation through dephosphorylation of lipopolysaccharide and adenosine triphosphate. Br J Pharmacol (2015) 172:4932–45. 10.1111/bph.13261 26222228 PMC4621995

[40] Davidson JA Urban T Tong S Twite M Woodruff A Wischmeyer PE Alkaline phosphatase, soluble extracellular adenine nucleotides, and adenosine production after infant cardiopulmonary bypass. PLoS One (2016) 11:e0158981. 10.1371/journal.pone.0158981 27384524 PMC4934870

[41] Mahmood A Engle MJ Alpers DH. Secreted intestinal surfactant-like particles interact with cell membranes and extracellular matrix proteins in rats. J Physiol (2002) 542:237–44. 10.1113/jphysiol.2002.017087 12096065 PMC2290410

[42] McConnell RE Higginbotham JN Shifrin DA Tabb DL Coffey RJ Tyska MJ. The enterocyte microvillus is a vesicle-generating organelle. J Cell Biol (2009) 185:1285–98. 10.1083/jcb.200902147 19564407 PMC2712962

[43] Akiba Y Mizumori M Guth PH Engel E Kaunitz JD. Duodenal brush border intestinal alkaline phosphatase activity affects bicarbonate secretion in rats. Am J Physiol Gastrointest Liver Physiol (2007) 293:G1223–33. 10.1152/ajpgi.00313.2007 17916646

[44] Šefčíková Z Hájek T Lenhardt L Racek L Mozes S. Different functional responsibility of the small intestine to high-fat/high-energy diet determined the expression of obesity-prone and obesity-resistant phenotypes in rats. Physiol Res (2008) 57:467–74. 17552870 10.33549/physiolres.931117

[45] Barbier de La Serre C Ellis CL Lee J Hartman AL Rutledge JC Raybould HE Propensity to high-fat diet-induced obesity in rats is associated with changes in the gut microbiota and gut inflammation. Am J Physiol Gastrointest Liver Physiol (2010) 299(2):G440–8. 10.1152/ajpgi.00098.2010 20508158 PMC2928532

[46] Malo MS A high level of intestinal alkaline phosphatase is protective against type 2 diabetes mellitus irrespective of obesity. EBioMedicine (2015) 2:2016–23. 10.1016/j.ebiom.2015.11.027 26844282 PMC4703762

[47] Kaliannan K Hamarneh SR Economopoulos KP Nasrin Alam S Moaven O Patel P Intestinal alkaline phosphatase prevents metabolic syndrome in mice. Proc Natl Acad Sci U S A (2013) 110:7003–8. 10.1073/pnas.1220180110 23569246 PMC3637741

[48] Bentala H Verweij WR Huizinga-Van der Vlag A van Loenen-Weemaes AM Meijer DKF Poelstra K. Removal of phosphate from lipid A as a strategy to detoxify lipopolysaccharide. Shock (2002) 18:561–6. 10.1097/01.shk.0000043623.17707.47 12462566

[49] Park BS Song DH Kim HM Choi B-S Lee H Lee J-O. The structural basis of lipopolysaccharide recognition by the TLR4-MD-2 complex. Nature (2009) 458:1191–5. 10.1038/nature07830 19252480

[50] Hoshino K Takeuchi O Kawai T Sanjo H Ogawa T Takeda Y Cutting edge: toll-like receptor 4 (TLR4)-deficient mice are hyporesponsive to lipopolysaccharide: evidence for TLR4 as the Lps gene product. J Immunol (1999) 162:3749–52. 10.1038/nri2275 10201887

[51] Akira S Yamamoto M Lipid a receptor TLR4-mediated signaling pathways. Adv Exp Med Biol (2009) 667:59–68. 10.1007/978-1-4419-1603-7_6 20665200

[52] Beutler B Rietschel ET. Innate immune sensing and its roots: the story of endotoxin. Nat Rev Immunol (2003) 3:169–76. 10.1038/nri1004 12563300

[53] Tuin A Poelstra K de Jager-Krikken A Bok L Raaben W Velders MP Role of alkaline phosphatase in colitis in man and rats. Gut (2009) 58:379–87. 10.1136/gut.2007.128868 18852260

[54] Bates JM Akerlund J Mittge E Guillemin K. Intestinal alkaline phosphatase detoxifies lipopolysaccharide and prevents inflammation in zebrafish in response to the gut microbiota. Cell Host Microbe (2007) 2:371–82. 10.1016/j.chom.2007.10.010 18078689 PMC2730374

[55] Ramasamy S Nguyen DD Eston MA Nasrin Alam S Moss AK Ebrahimi F Intestinal alkaline phosphatase has beneficial effects in mouse models of chronic colitis. Inflamm Bowel Dis (2011) 17:532–42. 10.1002/ibd.21377 20645323 PMC3154118

[56] Alam SN Yammine H Moaven O Ahmed R Moss AK Biswas B Intestinal alkaline phosphatase prevents antibiotic-induced susceptibility to enteric pathogens. Ann Surg (2014) 259:715–22. 10.1097/SLA.0b013e31828fae14 23598380 PMC3855644

[57] Heinzerling NP Liedel JL Welak SR Fredrich K Biesterveld BE Pritchard KA Intestinal alkaline phosphatase is protective to the preterm rat pup intestine. J Pediatr Surg (2014) 49:954–60; discussion 960. 10.1016/j.jpedsurg.2014.01.031 24888842 PMC4130394

[58] Rentea RM Liedel JL Welak SR Cassidy LD Mayer AN Pritchard KA Intestinal alkaline phosphatase administration in newborns is protective of gut barrier function in a neonatal necrotizing enterocolitis rat model. J Pediatr Surg (2012) 47(6):1135–41. 10.1016/j.jpedsurg.2012.03.018 22703783

[59] Biesterveld BE Koehler SM Heinzerling NP Rentea RM Fredrich K Welak SR Intestinal alkaline phosphatase to treat necrotizing enterocolitis. J Surg Res (2015) 196:235–40. 10.1016/j.jss.2015.02.030 25840489 PMC4578817

[60] Fawley J Koehler S Cabrera S Lam V Fredrich K Hessner M Intestinal alkaline phosphatase deficiency leads to dysbiosis and bacterial translocation in the newborn intestine. J Surg Res (2017) 218:35–42. 10.1016/j.jss.2017.03.049 28985873

[61] Malo MS Alam SN Mostafa G Zeller SJ Johnson PV Mohammad N Intestinal alkaline phosphatase preserves the normal homeostasis of gut microbiota. Gut (2010) 59:1476–84. 10.1136/gut.2010.211706 20947883

[62] Shifrin DA McConnell RE Nambiar R Higginbotham JN Coffey RJ Tyska MJ. Enterocyte microvillus-derived vesicles detoxify bacterial products and regulate epithelial-microbial interactions. Curr Biol (2012) 22:627–31. 10.1016/j.cub.2012.02.022 22386311 PMC3326206

[63] Martínez-Moya P Ortega-González M González R Anzola A Ocón B Hernández-Chirlaque C Exogenous alkaline phosphatase treatment complements endogenous enzyme protection in colonic inflammation and reduces bacterial translocation in rats. Pharmacol Res (2012) 66:144–53. 10.1016/j.phrs.2012.04.006 22569414

[64] Wang W Chen SW Zhu J Zuo S Ma YY Chen ZY Intestinal alkaline phosphatase inhibits the translocation of bacteria of gut-origin in mice with peritonitis: mechanism of action. PLoS One (2015) 10:e0124835. 10.1371/journal.pone.0124835 25946026 PMC4422672

[65] Yagi M Sakamoto K Inoue T Fukushima W Hashimoto T Shimizu K Effect of glutamine-enriched, elemental diet on regeneration of residual small bowel mucosa and hepatic steatosis following massive bowel resection. J Clin Biochem Nutr (1993) 15:219–25. 10.3164/jcbn.15.219

[66] Campbell EL Macmanus CF Kominsky DJ Keely S Glover LE Bowers BE Resolvin E1-induced intestinal alkaline phosphatase promotes resolution of inflammation through LPS detoxification. Proc Natl Acad Sci U S A (2010) 107:14303. 10.1073/pnas.0914730107 20660763 PMC2922533

[67] Liu W Hu D Huo H Zhang W Adiliaghdam F Morrison S Intestinal alkaline phosphatase regulates tight junction protein levels. J Am Coll Surg (2016) 222:1009–17. 10.1016/j.jamcollsurg.2015.12.006 27106638 PMC5684582

[68] Poelstra K Bakker WW Klok PA Kamps JA Hardonk MJ Meijer DK Dephosphorylation of endotoxin by alkaline phosphatase in vivo. Am J Pathol (1997) 151:1163–9. 9327750 PMC1858034

[69] Lei W Ni H Herington J Reese J Paria BC. Alkaline phosphatase protects lipopolysaccharide-induced early pregnancy defects in mice. PLoS One (2015) 10:e0123243. 10.1371/journal.pone.0123243 25910276 PMC4409290

[70] Bloch-Zupan A Hypophosphatasia: diagnosis and clinical signs – a dental surgeon perspective. Int J Paediatr Dent (2016) 26(6):426–38. 10.1111/ipd.12232 27030892

[71] Orimo H. Pathophysiology of hypophosphatasia and the potential role of asfotase alfa. Ther Clin Risk Manag (2016) 12:777–86. 10.2147/TCRM.S87956 27274262 PMC4876073

[72] Whyte MP Rockman-Greenberg C Ozono K Riese R Moseley S Melian A Asfotase alfa treatment improves survival for perinatal and infantile hypophosphatasia. J Clin Endocrinol Metab (2016) 101:334–42. 10.1210/jc.2015-3462 26529632 PMC4701846

[73] Hofmann C Seefried L Jakob F. Asfotase alfa: enzyme replacement for the treatment of bone disease in hypophosphatasia. Drugs Today (Barc) (2016) 52:271–85. 10.1358/dot.2016.52.5.2482878 27376160

[74] Peters E van Elsas A Heemskerk S Jonk L van der Hoeven J Arend J Alkaline phosphatase as a treatment of sepsis-associated acute kidney injury. J Pharmacol Exp Ther (2013) 344:2–7. 10.1124/jpet.112.198226 23131595

[75] Heemskerk S Masereeuw R Moesker O Bouw MP van der Hoeven JG Peters WH Alkaline phosphatase treatment improves renal function in severe sepsis or septic shock patients. Crit Care Med (2009) 37:417–423,e1. 10.1097/CCM.0b013e31819598af 19114895

[76] Lukas M Drastich P Konecny M Gionchetti P Urban O Cantoni F Exogenous alkaline phosphatase for the treatment of patients with moderate to severe ulcerative colitis. Inflamm Bowel Dis (2010) 16:1180–6. 10.1002/ibd.21161 19885903

[77] Rader BA Kremer N Apicella MA Goldman WE McFall-Ngai MJ. Modulation of symbiont lipid a signaling by host alkaline phosphatases in the squid-vibrio symbiosis. MBio (2012) 3. 10.1128/mBio.00093-12 22550038 PMC3569863

[78] McFall-Ngai M Hadfield MG Bosch TCG Carey HV Domazet-Lošo T Douglas AE Animals in a bacterial world, a new imperative for the life sciences. Proc Natl Acad Sci U S A (2013) 110:3229–36. 10.1073/pnas.1218525110 23391737 PMC3587249

[79] Robison R Soames KM The possible significance of hexosephosphoric esters in ossification. Biochem J (1924) 18:740–54. 10.1097/00003086-199106000-00001 16743453 PMC1263971

